# Abdominal aortic calcification score among several vascular calcification scores of plain radiograph is the most reliable predictor of severe coronary artery calcification in dialysis patients

**DOI:** 10.1080/0886022X.2017.1398666

**Published:** 2017-11-20

**Authors:** Su Mi Lee, Hye Won Lee, Young Ki Son, Seong Eun Kim, Won Suk An

**Affiliations:** Department of Internal Medicine, Dong-A University, Busan, Republic of Korea

**Keywords:** Aortic calcification, bone mineral density, coronary calcification, dialysis, vascular calcification

## Abstract

**Aim:** Coronary artery calcification (CAC) score on computed tomography (CT) or vascular calcification (VC) scores on plain radiographs are associated with cardiovascular events and fracture. We investigated which VC score among several VC scores on plain radiographs is predictor of CAC, and whether VC scores are related with bone mineral density (BMD) in dialysis patients.

**Methods:** We checked several plain radiographs (hands and pelvis [HP], feet and lateral lumbar spine), BMD and multidetector CT scans of 55 patients maintaining dialysis in this cross-sectional study. We analyzed data to find predictors for severe CAC which was defined as CAC scores >400 on CT.

**Results:** Patients with severe CAC on CT had a higher proportion of abdominal aortic calcification (AAC) score ≥5, HP score ≥3 and feet ≥1 than those without severe CAC. The CAC score on CT was positively correlated with all VC scores on plain radiographs. The AAC and CAC scores were negatively correlated with T-scores for the BMD at the forearm and positively correlated with osteoprotegerin levels. Among several VC scores on plain radiographs, the AAC ≥5 were independently associated with severe CAC on CT.

**Conclusions:** Several plain radiographs evaluating VC scores, including a lateral lumbar spine view at the very least, can replace CT checking CAC score in dialysis patients. The AAC score ≥5 may not only reveal severe CAC but also give a hint of low bone mass at the forearm.

## Introduction

Patients on dialysis are at particularly high risk for death, and cardiac disease is the major cause of death. Vascular calcification (VC) in dialysis patients is significantly associated with an increase in cardiovascular morbidity and mortality. A strong correlation between the coronary artery calcification (CAC) score on computed tomography (CT) and coronary artery disease (CAD) has been shown in several studies, but radiation exposure and cost still pose a problem [1,2]. Plain radiography is relatively cheap and offers a simple method for evaluating VC. Previous studies have reported that VC scores on plain radiographs are associated with cardiac events in dialysis patients [3–7]. It has also been illustrated that high or meaningful VC scores on plain radiographs may act as a predictor of cardiac calcification or stenosis before coronary angiography in dialysis patients [8]. One previous study showed that the abdominal aortic calcification (AAC) score on plain radiographs had a very good correlation with the identification of CAC on CT [9]. The other study also showed that both lateral abdominal and pelvic plain radiographs are acceptable alternatives to CT in evaluating VC [10]. However, no study has investigated which VC score among several VC scores on plain radiographs is useful predictor for CAC on CT.

Bone mineral density (BMD) using dual X-ray absorptiometry (DEXA) is a useful method for evaluating bone mass [11–13]. Low BMD frequently occurs in dialysis patients, and it increases the risk of fracture [14,15]. VC of superficial femoral arteries on CT has been inversely correlated with the femoral T-score for BMD in patients with chronic kidney disease (CKD) [11]. The AAC on plain radiographs was definitely related with a low BMD in a population without CKD [16]. However, no study has estimated whether the VC score on plain radiographs are correlated with the T-score for BMD in dialysis patients.

Here, we examined which VC score among several VC scores on plain radiographs was the most reliable predictor of CAC on CT in dialysis patients. We also investigated whether VC scores on plain radiographs were related with the T-score on BMD.

## Methods

### Study design and patients

We conducted a cross-sectional study at the Dong-A University Dialysis Center from March 2013 to September 2014. Patients who had been receiving dialysis for at least 6 months and who were over 20 years of age were enrolled. The exclusion criteria were as follows: patients with a history of dialysis modality change, a history of active infection within the past 3 months or a history of kidney transplantation. Enrolled patients were first assessed using plain radiographs (including the hands and pelvis [HP], feet and lateral lumbar spine views), BMD using DEXA and multidetector CT (MDCT). Hemodialysis (HD) patients received bicarbonate-based dialysate and polysulfone dialyzers (Fresenius, Bad Homburg, Germany) three times weekly. Peritoneal dialysis (PD) patients received four exchanges per day using a standard regimen (8 L/d).

### Laboratory measurements

Routine laboratory tests using fasting blood samples were performed before dialysis. Serum levels of hemoglobin, glucose, blood urea nitrogen, creatinine, albumin, calcium, phosphorus, alkaline phosphatase (ALP), C-reactive protein, parathyroid hormone (PTH), total cholesterol, triglycerides, high-density lipoprotein cholesterol and low-density lipoprotein cholesterol were analyzed. Serum 25-hydroxyvitamin D and 1,25-dihydroxyvitamin D levels were assessed using a radioimmunoassay kit (DiaSorin Inc., Stillwater, MN). Fetuin-A, osteoprotegerin (OPG) and receptor activator of nuclear factor-κB ligand (RANKL) levels were analyzed with enzyme-linked immunosorbent assay (ELISA, BioVendor Laboratory Medicine, Modrice, Czech Republic). We additionally measured fibroblast growth factor 23 (FGF-23) levels using a Millipore FGF23 ELISA Kit (Millipore Co., Billerica, MA).

### Vascular calcification score on plain radiographs

Plain radiographs of the lateral lumbar spine view for AAC, HP and the feet were evaluated. The VC scores were estimated using previously reported methods [3,4,17,18]. A meaningful VC score was defined as following criteria: AAC score ≥5; HP score ≥3; and the presence of arterial media calcification on the feet [3–6]. The VC scores were individually determined by two nephrologists, and a consensus was reached on the interpretation of all radiographs.

### Coronary artery calcification score on multidetector computed tomography

A 320-detector-row scanner (Aquilion ONE, Toshiba Medical Systems, Otawara, Japan) was used to evaluate the CAC score of each lesion; the left main, left anterior descending, left circumflex and right coronary arteries were calculated with the Agatston method. A radiologist who was blinded to the participant’s age, sex and name performed the scoring of the CAC. The total Agatston scores (TAS) > 400 was defined as severe CAC [19].

### Bone mineral density

BMD was measured at the lumbar spine (L1–L4), the hip at the femur neck and the total hip, and the forearm at the radius and ulna by means of DEXA using a Discovery W scanner (Hologic Inc., Waltham, MA). BMD results were obtained as T-score; a T-score is the number of SDs from the mean of a healthy young adult population (20–40 years old). Osteoporosis was defined as a BMD that was 2.5 SD or more below the normal level for healthy young adults.

### Statistical analysis

Data were expressed as mean ± SD and medians (interquartile ranges), depending on the variable distribution, and Student’s *t*-test and Mann–Whitney *U*-test were analyzed as appropriate. Categorical variables were expressed as frequency, and the Chi-squared test was used for comparison. Pearson’s correlation coefficient was applied to identify the correlation between the degree of CAC on CT and any related parameters. The area under the receiver operating characteristic (ROC) curve (AUC) was used for the prediction of diagnostic performance of VC scores. Univariate regression analysis was conducted to identify whether CAC on CT was related to several VC scores on plain radiographs. To evaluate the factors independently associated with the degree of CAC on CT, multivariate logistic regression analysis was performed. A *p* values <.05 was considered to be statistically significant. All statistical calculations were performed using the Statistical Package for the Social Sciences (SPSS) version 18.0 (SPSS Inc., Chicago, IL).

## Results

### Clinical characteristics in accordance with severe CAC

Fifty-five dialysis patients were enrolled in the study. The baseline characteristics of these participants are shown in Table 1. The mean age was 58.3 ± 10.4 years, and 24 patients (43.6%) were male. Out of all the patients, 35 (63.6%) received HD and the remaining 20 (36.4%) received PD.

**Table 1.** Comparison of clinical characteristics in accordance with the degree of coronary artery calcification score on computed tomography.

Characteristics	Total (*n* = 55)	CACS <400 (*n* = 35)	CACS >400 (*n* = 20)	*p* value
Age (years)	58.3 ± 10.4	56.2 ± 11.3	62.0 ± 7.3	.045
Male, *n* (%)	24 (43.6)	15 (42.9)	9 (45.0)	.877
HD (vs. PD), *n* (%)	35 (63.6)	22 (62.9)	13 (65.0)	.874
Duration (months), *n* (%)	51.1 ± 37.9	43.0 ± 33.1	65.2 ± 42.4	.036
Diabetes mellitus, *n* (%)	29 (52.7)	15 (42.9)	14 (70.0)	.052
Body mass index (kg/m^2^)	23.0 ± 3.3	23.2 ± 3.2	22.6 ± 3.5	.561
Calcification scores on plain radiograph, *n* (%)				
Abdominal aorta ≥5, *n* (%)	28 (50.9)	11 (31.4)	17 (85.0)	<.001
Hands and pelvis ≥3, *n* (%)	29 (52.7)	14 (40.0)	15 (75.0)	.012
Feet ≥1, *n* (%)	28 (50.9)	14 (40.0)	14 (70.0)	.032
Total Agatston score*	255 (81–1172)	96 (7–216)	1832 (1112–2487)	<.001
Calcium (mg/dL)	9.0 ± 0.7	8.9 ± 0.8	9.2 ± 0.6	.156
Phosphorus (mg/dL)	5.5 ± 1.5	5.5 ± 1.5	5.5 ± 1.7	.954
Alkaline phosphatase (IU/L)	285.2 ± 122.2	260.8 ± 107.8	327.8 ± 136.5	.050
C-reactive protein (mg/dL)	0.4 ± 0.9	0.4 ± 1.1	0.4 ± 0.4	.869
Total cholesterol (mg/dL)	158.2 ± 33.6	160.8 ± 32.1	153.6 ± 36.4	.450
Triglyceride (mg/dL)	141.2 ± 76.2	135.1 ± 69.6	151.8 ± 87.4	.441
HDL (mg/dL)	42.7 ± 11.1	44.9 ± 11.0	38.9 ± 10.5	.054
LDL (mg/dL)	94.2 ± 29.4	96.0 ± 28.5	91.0 ± 31.5	.551
PTH (pg/mL)	350.7 ± 224.4	326.1 ± 197.8	393.6 ± 264.7	.288
1,25(OH)_2_D (pg/mL)	21.8 ± 10.0	22.1 ± 9.9	21.2 ± 10.4	.763
25(OH)D (ng/mL)	13.3 ± 6.4	13.6 ± 6.2	12.6 ± 6.9	.590
Fetuin-A (μg/mL)	209.3 ± 50.9	208.5 ± 53.9	210.7 ± 46.3	.896
FGF-23 (pg/mL)*	1683 (360–2894)	1045 (214–2807)	2677 (1612–3812)	.112
Osteoprotegerin (pmol/L)	22.7 ± 8.6	20.5 ± 7.9	26.9 ± 8.4	.018
RANKL (pmol/L)*	263.1 (138.7–404.3)	322 (134–436)	215 (139–329)	.414

HD: hemodialysis; PD: peritoneal dialysis; HDL: high-density lipoprotein cholesterol; LDL: low-density lipoprotein cholesterol; PTH: parathyroid hormone; 1,25(OH)_2_D: 1,25-dihydroxyvitamin D; 25(OH)D: 25-hydroxyvitamin D; FGF-23: fibroblast growth factor 23; RANKL: receptor activator of nuclear factor-κB ligand; CACS: coronary artery calcification score.

* Data are expressed as median (interquartile range) and the Mann–Whitney *U*-test was used for comparison.

We classified patients into two groups in accordance with the degree of CAC: patients with severe CAC (TAS >400) or those without severe CAC (Table 1). The proportions of patients with AAC score ≥5, HP score ≥3 and the presence of feet calcification were significantly higher for those with severe CAC than those without severe CAC. Among several bone markers, the OPG and ALP levels were significantly higher in patients with severe CAC than in those without severe CAC. However, no significant differences in the laboratory findings, including PTH, fetuin-A, FGF-23 and RANKL, were found between the two groups.

We identified the diagnostic performance of the AAC and HP scores for the prediction of severe CAC on CT using ROC analysis. The AUCs for the AAC and HP scores were 0.815 (95% confidence interval [95% CI] = 0.694–0.936, *p <* .001) and 0.798 (95% CI = 0.684–0.921, *p<* .001), respectively. The VC scores of the AAC ≥5 predicted severe CAC on CT with 82.5% sensitivity and 65.0% specificity and HP ≥3 predicted severe CAC on CT with and 72.9% sensitivity and 68.6% specificity.

### Association between VC scores on plain radiographs and T-score on BMD

**Figure 1.**
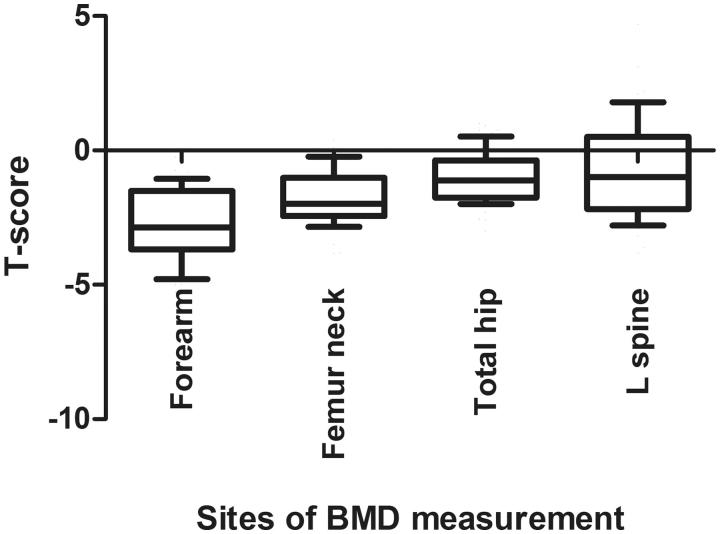
T-score at sites of bone mineral density measurement.

We compared the mean T-score on BMD according to the sites where it was assessed (Figure 1). The mean T-score for BMD measured at the forearm were relatively lower than that for the BMD assessed at the femur neck, total hip or lumbar spine (−2.8 ± 1.4, −1.7 ± 1.0, −1.0 ± 0.9 and −0.7 ± 1.8, respectively). T-score for the BMD measurements taken at the forearm, total hip and femur neck in those with an AAC score ≥5 were significantly lower as compared with patients with an AAC score <5; however, there were no significant differences in T-score for the BMD of the lumbar spine in the two groups (Table 2). There were also no significant differences in T-score of the BMD assessed at the forearm, total hip, femur neck and lumbar spine between the VC score of the HP ≥3 and <3 groups. In patients with arterial media calcification of the feet, T-score of the BMD taken at the femur neck and the lumbar spine were significantly higher than those in the other group.

**Table 2.** Comparison of t-score on sites of BMD measurement in accordance with vascular calcification on plain radiographs.

T-score on BMD	AAC <5	AAC ≥5	*p* value	HP <3	HP ≥3	*p* value	Feet <1	Feet ≥1	*p* value
Forearm	−2.3 ± 1.2	−3.3 ± 1.4	.004	−2.8 ± 1.1	−2.8 ± 1.6	.978	−3.1 ± 1.1	−2.5 ± 1.5	.057
Total hip	−0.7 ± 0.8	−1.3 ± 1.0	.024	−1.0 ± 0.8	−1.0 ± 1.0	.806	−1.2 ± 0.7	−1.2 ± 0.7	.173
Femur neck	−1.4 ± 0.9	−2.1 ± 1.0	.009	−1.6 ± 0.9	−1.8 ± 1.1	.477	−2.0 ± 0.8	−1.4 ± 1.1	.035
Lumbar spine	−0.5 ± 1.6	−1.0 ± 1.8	.299	−1.1 ± 1.6	−0.5 ± 1.8	.172	−1.3 ± 1.4	−0.2 ± 1.9	.013

BMD: bone mineral density; AAC: abdominal aorta calcification; HP: hands and pelvis.

Thirty-three (60.0%) patients showed osteoporosis in the forearm. We identified the diagnostic performance of AAC for the prediction of osteoporosis in the forearm. The AUCs for the AAC score were 0.668 (95% CI = 0.529–0.808, *p* = .028). A VC score that was AAC ≥5 predicted osteoporosis in the forearm with 59.8% sensitivity and 68.8% specificity.

### Correlation between the AAC score on plain radiographs or CAC score on CT and related parameters

A significant correlation was observed between the degree of AAC on plain radiographs or CAC on CT and related parameters (Table 3). The AAC score was significantly correlated with age, dialysis vintage, HP score, T-score for the BMD at the forearm, FGF-23 level and OPG level. However, there was no significant correlation between the T-score of the BMD for the total hip, femur neck and lumbar spine and the AAC score.

**Table 3.** Correlation between degrees of abdominal aorta calcification score on plain radiograph or coronary artery calcification on computed tomography and related parameters.

	AAC score		CAC score on CT	
Characteristics	*r*	*p* value	*r*	*p* value
Age (years)	0.390	.002	0.329	.014
Duration (months)	0.464	<.001	0.294	.029
Calcification scores on plain radiograph				
Abdominal aorta			0.543	<.001
Hands and pelvis	0.271	.037	0.548	<.001
Feet	0.079	.547	0.268	.048
T-score on bone mineral density				
Forearm	−0.273	.035	−0.304	.024
Total hip	−0.125	.340	−0.201	.141
Femur neck	−0.226	.131	−0.245	.113
Lumbar spine	−0.090	.494	−0.027	.843
Alkaline phosphatase (IU/L)	0.019	.883	0.273	.044
HDL (mg/dL)	−0.102	.458	−0.317	.018
PTH (pg/mL)	0.127	.333	0.004	.979
1,25(OH)_2_D (pg/mL)	0.238	.071	0.023	.873
25(OH)D (ng/mL)	0.218	.098	0.126	.363
Fetuin-A (μg/mL)	−0.119	.437	0.085	.590
FGF-23 (pg/mL)	0.401	.013	0.211	.209
Osteoprotegerin (pmol/L)	0.296	.048	0.383	.011
RANKL (pmol/L)	−0.068	.677	−0.157	.346

HDL: high-density lipoprotein cholesterol; PTH: parathyroid hormone; 1,25(OH)_2_D: 1,25-dihydroxyvitamin D; 25(OH)D: 25-hydroxyvitamin D; FGF-23: fibroblast growth factor 23; RANKL: receptor activator of nuclear factor-κB ligand; AAC: abdominal aorta calcification; CAC: coronary artery calcification; CT: computed tomography.

The CAC score on CT was positively correlated with age, dialysis vintage, OPG level and VC scores on plain radiographs, such as AAC, HP and feet. In addition, the T-score for the BMD measured at the forearm had a significantly negative association with the presence of CAC on CT, but this finding was not observed for the T-score of the BMD measured at the total hip, femur neck or lumbar spine. A significant correlation between the CACs on CT and several bone markers other than OPG, such as FGF-23, fetuin-A and RANKL, was not observed.

### Independent factors associated with severe CAC on CT

Upon univariate regression analysis, dialysis vintage, OPG level, AAC ≥5, HP ≥3 and feet ≥1 were associated with severe CAC on CT. To confirm the predictors for severe CAC seen on CT, we performed multiple logistic regression analysis (Table 4). Among several VC scores on plain radiographs, the AAC score ≥5 were independently associated with higher rates of severe CAC on CT after adjustment for age, DM, dialysis vintage, OPG level and HP ≥3 and feet ≥1. Dialysis vintage was also independent factor associated with severe CAC on CT.

**Table 4.** Independent factors associated with severe coronary artery calcification on computed tomography.

	Relative risk (95% CI)			
Variables	Univariate	*p* value	Multivariate	*p* value
Age (years)	1.063 (0.999–1.130)	.052	0.912 (0.783–1.062)	.235
Diabetes mellitus, *n* (%)	3.111 (0.968–9.998)	.057	16.812 (0.722–391.49)	.079
Duration (months)	1.016 (1.000–1.032)	.046	1.050 (1.003–1.099)	.036
HDL (mg/dL)	0.947 (0.895–1.002)	.061	0.845 (0.714–1.001)	.051
Osteoprotegerin (pmol/L)	1.102 (1.010–1.202)	.029	1.098 (0.937–1.287)	.247
Calcification scores on plain radiograph				
Abdominal aorta ≥5	12.364 (2.989–51.138)	.001	14.829 (1.215–180.99)	.035
Hands and pelvis ≥3	4.500 (1.332–15.201)	.015	4.210 (0.344–51.486)	.261
Feet ≥1	3.500 (1.085–11.292)	.036	0.484 (0.050–4.636)	.529

HDL: high-density lipoprotein cholesterol.

## Discussion

Electron-beam CT or MDCT are representative, noninvasive methods for assessing the presence and severity of CAD [2]. Calcification scores are quantified by computer-based analysis, and good correlation between CAC and cardiac events has been shown in several studies [1,20,21]. Dialysis patients with CAC score >400 showed the highest mortality rate and cardiovascular event rate [22]. Previous studies just suspected that VC scores on plain radiographs related with cardiovascular events were associated with CAC on CT. This study reconfirmed that AAC, HP and feet scores on plain radiographs were associated with a higher likelihood of CAC on MDCT. Based on our data, physicians may assume the possibility of having CAC, if patients treated with dialysis have meaningful VC scores on plain radiographs. To our knowledge, this is the first report to show AAC score ≥5 was an independent predictor for severe CAC score by comparing several VC scores on plain radiographs. Several plain radiographs obtained from a variety of locations will be useful for evaluating CAC without overlooking VC, if we do not check MDCT. However, lateral view of lumbar spine should be checked if we have to choose only one plain radiographs.

An association between VC and osteoporosis has been reported in previous studies [16]. Low bone mass and fracture are important complications in dialysis patients. The association between low bone mass and fractures in the general population is well recognized [23], but the result is not consistent in dialysis patients [24]. VC is positively associated with vertebral fractures, and it is inversely correlated with BMD [16]. The risk of spine and hip fractures increases with a lower BMD at the spine and hip in patients with renal dysfunction [15]. On the other hand, several studies have reported that there is no relationship between hip or spine BMD and fractures [25,26]. Jamal et al. found that the wrist BMD was low in patients with fractures, but hip or spine BMD was not related to a patient’s fracture risk [25]. Our study indicated that dialysis patients with an AAC score ≥5 have a higher possibility of being diagnosed with a low forearm BMD. AAC score ≥5 among other VC scores on plain radiographs is the most reliable tool for considering low BMD of femur neck and total hip. These results partly imply that an AAC score ≥5 on plain radiographs indirectly indicates low bone mass and possible fracture risk in dialysis patients. Although spine bone density is higher than the bone density of the radius or hip in CKD stage 5, AAC was not related with BMD of lumbar spine in our study [27]. It is doubtful whether high density of aortic calcification may mask low bone mass of conventional BMD of lumbar spine, especially in patients with AAC. Further studies will be necessary to determine the role of the AAC score on plain radiograph as a predictor for fracture risk in dialysis patients.

In this study, the BMD at the forearm was relatively lower than the BMD at the femur neck, total hip or lumbar spine. In dialysis patients, the forearm BMD typically is lower than the spine BMD, and the spine BMD may overestimate the overall bone density [28]. This discrepancy may be explained by the fact that the forearm contains a mixture of trabecular and cortical bone, and the spine BMD is often hindered by artifacts, such as VC. Because the BMD measurements are typically obtained using DEXA, a two-dimensional method, it is difficult to assess the precise bone mass if an artifact is present on the pass of the x-ray beam. A variety of artifacts, such as VC, peritoneal fluid, calcium tablets in the intestine and scoliosis, can generate an error in the interpretation of the BMD in dialysis patients. However, even if the results of the BMD in dialysis patients are questionable, DEXA is still a useful method for the diagnosis of bone loss and the prediction of fracture [13]. Unexpectedly, T-score for the BMD were inversely presented in comparison with the absence or presence of arterial media calcification on the feet compared with the AAC in this study. Patients with feet calcifications have a greater tendency toward T-score when BMD is measured at the forearm. Different pathogenic mechanisms may be related to depending on where the VC has been located. Further studies will be needed to identify any cross-linkages between VC and bone mass on BMD.

Several markers, such as OPG, RANKL, fetuin-A and FGF-23, have been reported to act as VC inhibitors or inducers, but it is not clear which factor is most influential for the VC. Previous studies have reported that OPG levels are positively associated with CAC on CT, leading to cardiovascular disease in dialysis patients as well as the general population [29–32]. In our study, dialysis patients with severe CAC on CT or meaningful VC on plain radiographs had higher OPG levels than those without severe CAC or meaningful VC. Based on these results, we suspect that high circulating OPG levels act to reduce bone resorption and induce adynamic bone disease in dialysis patients. In contrast, calcium and phosphorous not in-fluxing into the bone can be easily integrated into osteoblast-like endothelial cells and induce VC through decreased bone resorption in dialysis patients. Fetuin-A is synthesized by the liver, and it may act as an inhibitor of VC by forming a solution of calcium phosphate salt. In previous studies, a low fetuin-A level has been associated with a high incidence of CAC, and it is also related with an increased risk for cardiovascular mortality in dialysis patients [33,34]. However, we did not observe a significant association between fetuin-A and CAC. Further studies will be needed to evaluate the role of several biomarkers for predicting VC and subsequent mortality in dialysis patients.

This study had some limitations. First, the power of the study was limited because of the relatively small number of participants. Second, because a history of fracture was present in only six patients, we cannot identify the relationship between bone mass and fracture risk. Long-term studies will be needed to predict the risk of fracture using biomarkers or BMD in dialysis patients. Despite these limitations, this study demonstrated that the meaningful VC scores obtained using several plain radiographs were related with CAC on CT in patients receiving dialysis. In addition, we found that an AAC score was the most reliable predictor of severe CAC on CT and maybe estimate low T-score on BMD at the forearm among several VC scores on plain radiograph. This is the first study to investigate the association between VC on plain radiographs and T-score on BMD.

## Conclusion

Several plain radiographs evaluating VC scores, including a lateral lumbar spine view at the very least, can replace MDCT checking CAC score in dialysis patients. When identified on plain radiographs, the AAC score ≥5 may not only reveal severe CAC, which indicates higher cardiovascular events, but also gives a hint of low bone mass at the forearm, suggesting a possible risk of fracture.
